# Microbiome–Metabolome Crosstalk in HPV Pathogenesis: From Ecosystem Dynamics to Translational Biomarkers

**DOI:** 10.34133/csbj.0158

**Published:** 2026-07-03

**Authors:** Mariano A. Molina, Wenkui Dai

**Affiliations:** ^1^ Department of Laboratory Medicine, Karolinska Institutet, ANA Futura, Huddinge, Sweden.; ^2^ Department of Cellular Therapy and Allogeneic Stem Cell Transplantation (CAST), Karolinska University Hospital, Huddinge, Sweden.; ^3^ Department of Obstetrics and Gynecology, Peking University Shenzhen Hospital, Shenzhen, China.; ^4^ Institute of Obstetrics and Gynecology, Shenzhen PKU-HKUST Medical Center, Shenzhen, China.; ^5^ Shenzhen Key Laboratory on Technology for Early Diagnosis of Major Gynecologic Diseases, Shenzhen, China.

## Abstract

High-risk human papillomavirus (hrHPV) infection alters the cervicovaginal microenvironment, driving metabolic reprogramming that influences viral persistence and progression to cervical cancer. This review adopts a systems-level perspective to synthesize findings from recent metabolomic studies across urine, vaginal swabs, and cervicovaginal fluids, highlighting consistent trends from cervicovaginal health through hrHPV infection, persistence, cervical lesion development, and cancer. HPV infection is characterized by increased microbial amines and oxidative stress, whereas viral persistence and high-grade cervical lesions exhibit disrupted metabolism of amino acids, lipids, and nucleotides. Cervical cancer is associated with distinct metabolic signatures involving sphingolipids, ketone bodies, and intermediates of the tricarboxylic acid cycle. Collectively, metabolic profiles emerge as functional readouts of host–microbiome interactions, often showing stronger associations with clinical outcomes than microbial composition alone. Integrative multiomics approaches combining metabolomics with microbiome- and host-derived data are beginning to uncover coordinated biological pathways underlying HPV pathogenesis and may improve risk stratification and biomarker discovery. Despite methodological heterogeneity, converging evidence supports the potential of metabolic profiling for early detection of cervical neoplasia and stratification of hrHPV-positive women, although reproducibility across studies remains limited. Future longitudinal and integrative studies, supported by standardized analytical frameworks and computational modeling, are needed to clarify causal mechanisms and enable the development of clinically actionable biomarkers and targeted interventions.

## Introduction

A persistent infection with high-risk human papillomavirus (hrHPV), particularly HPV16 and HPV18, is the main etiological factor of virtually all cervical cancer cases [1,2]. While hrHPV infections are highly prevalent among sexually active individuals, most infections are transient and are cleared by the immune system within 1 to 2 years [3]. Only a minority of infections progress to cervical intraepithelial neoplasia (CIN) and, in some cases, invasive cervical carcinoma (ICC) [3,4]. Persistent hrHPV infection is driven by the expression of the viral oncogenes E6 and E7, which disrupt key tumor suppressor pathways through the degradation of p53 and inactivation of retinoblastoma protein, respectively [3]. These alterations promote cell-cycle dysregulation, genomic instability, immune evasion, and progressive epithelial transformation [3,5]. Over time, persistent infection may lead to the development of cervical precancer and invasive cervical cancer. Notably, HPV acquisition and reactivation may also occur in pre- and postmenopausal women, likely reflecting age-related immune modulation, hormonal changes, and alterations in the cervicovaginal microenvironment [6,7]. This suggests that host, viral, and microenvironmental factors collectively influence the course and outcomes of hrHPV infections in the cervix [8]. Understanding the mechanisms underlying hrHPV persistence, and how these relate to the development of precancerous lesions and cancer, is therefore critical for the development of more accurate biomarkers, improved risk stratification tools, and novel therapeutic strategies [9,10].

The cervicovaginal microenvironment is a complex ecosystem containing epithelial cells, immune mediators, mucins, commensal and pathogenic microbes, and a diverse array of metabolites [11]. This microenvironment is crucial for host defenses against pathogens and mucosal homeostasis, but it can be profoundly altered by viral infections [12–15]. Metabolites, small-molecular intermediates and end products of cellular processes, are both reflective and modulatory of the biological state [16]. As such, the study of chemical processes involving metabolites, small-molecule substrates, intermediates, and products of cellular metabolism (metabolomics) offers a snapshot of the functional output of the microenvironment, providing insights into host–pathogen–microbiome interactions at the biochemical level [17]. Changes in local metabolite concentrations can also influence viral replication, immune activation, and epithelial transformation, making the cervicovaginal metabolome a valuable tool to assess hrHPV pathogenesis [18,19].

Within the cervicovaginal niche, metabolites arise from host epithelial and immune cells, resident microbes, and their interactions, collectively shaping mucosal function [20]. Commonly detected metabolites include organic acids (e.g., lactic acid and acetate), carbohydrates and their derivatives (e.g., glycogen and glucose-6-phosphate), amino acids and N-acetylated amino acids, polyamines, lipids and phospholipids, nucleotides, and microbially derived byproducts including biogenic amines and short-chain fatty acids (SCFAs) [21–25]. Together, these compounds reflect key biological processes such as epithelial barrier maintenance, immune regulation, microbial metabolism, and oxidative stress. Most cervicovaginal metabolomic studies rely on mass-spectrometry-based approaches, primarily liquid chromatography–mass spectrometry (LC–MS) and gas chromatography–mass spectrometry (GC–MS), applied to vaginal swabs, cervicovaginal lavage (CVL), vaginal secretions, or urine, enabling broad, untargeted profiling of metabolic changes across disease states [26]. LC–MS offers broader coverage of polar and lipid metabolites, whereas GC–MS provides robust quantification of volatile and derivatized metabolites with a higher chromatographic resolution [27,28]. Emerging technologies such as laser-assisted rapid evaporative ionization mass spectrometry (LA-REIMS) further allow rapid metabolic phenotyping directly from cytological samples [29,30]. While these platforms differ in metabolite coverage, sensitivity, and sample requirements, they collectively provide complementary insights into the biochemical landscape of the cervicovaginal niche and its perturbation during hrHPV infection and disease progression.

Over the past few years, a growing number of studies have applied these mass spectrometry technologies to a variety of cervicovaginal and systemic sample types to delineate metabolic signatures associated with hrHPV infection status, cervical lesion severity, and treatment response [31–33]. Recent cross-sectional and longitudinal studies have identified widespread metabolic alterations associated with hrHPV infection, cervical lesion progression, and treatment response, affecting multiple host and microbial metabolic pathways [31–34]. However, a comprehensive analysis of the temporal changes that occur upon hrHPV infection and related disease is still lacking, and an in-depth assessment of these reports is necessary. To this end, this review synthesizes and critically examines the metabolomic landscape of the cervicovaginal environment across the spectrum of hrHPV pathogenesis. The review focuses on identifying recurring metabolic alterations in cervicovaginal health, as well as in early hrHPV infection, viral persistence and clearance, low- and high-grade squamous intraepithelial lesions (LSIL and HSIL), and cervical cancer. This review also highlights areas of divergence between studies, examines methodological and contextual limitations, and suggests directions for future research. The relevant literature was identified through searches of PubMed and Web of Science using combinations of keywords related to hrHPV, cervicovaginal metabolomics, microbiome, cervical cancer, and multiomics. Ultimately, this review aims to provide a conceptual framework for integrating metabolomics into cervical cancer prevention and control strategies.

## Microbiome–Metabolome Interactions in HPV Pathogenesis

The cervicovaginal microenvironment is shaped by a dynamic interplay between resident microbes and the local metabolome, with substantial consequences for epithelial health, immunity, and hrHPV infection outcomes [34–38]. Dominance by *Lactobacillus* species, particularly *Lactobacillus crispatus*, is associated with a stable, acidic environment (pH 3.8 to 4.5) enriched in protective metabolites such as lactic acid and indole derivatives, which suppress dysbiotic taxa, reinforce epithelial barrier integrity, and promote anti-inflammatory signaling that may limit hrHPV persistence [39,40]. Conversely, microbial dysbiosis, characterized by the overgrowth of anaerobic taxa such as *Gardnerella vaginalis*, *Prevotella timonensis*, *Fannyhessea vaginae*, and *Megasphaera*, is linked to shifts in metabolite profiles that favor hrHPV persistence and lesion progression [35,41–43].

**Fig. 1.**
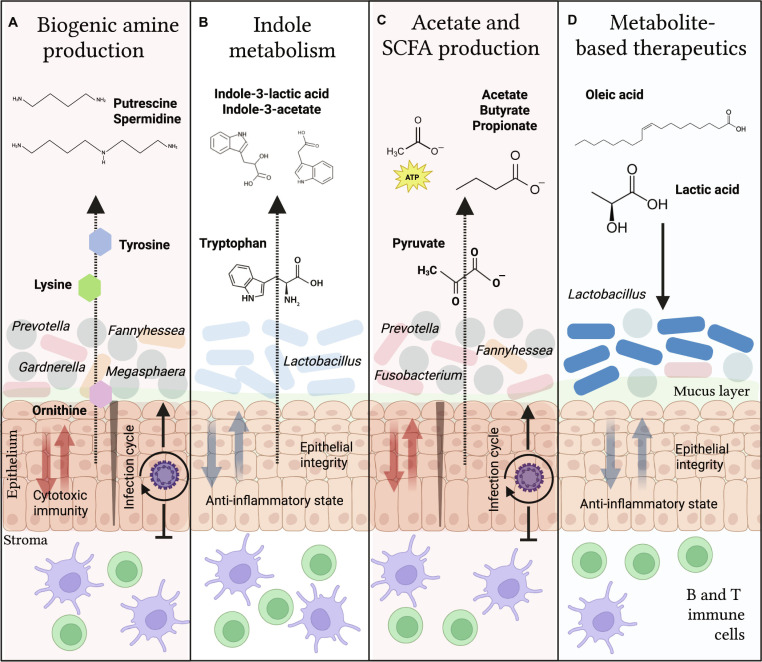
Microbiome-driven metabolic pathways shaping HPV pathogenesis and therapeutic opportunities. This schematic illustrates key cervicovaginal microbial metabolic pathways that influence HPV infection dynamics and epithelial health. (A) Dysbiotic bacteria (e.g., *Prevotella*, *Gardnerella*, *Fannyhessea*, and *Megasphaera*) decarboxylate amino acids (ornithine, lysine, and tyrosine) to produce biogenic amines (e.g., putrescine and spermidine), which can disrupt epithelial integrity and promote viral persistence. (B) Commensal *Lactobacillus* convert tryptophan into indole derivatives (e.g., indole-3-lactic acid and indole-3-acetate), thereby fostering an anti-inflammatory environment and protecting the epithelial barrier. (C) Anaerobes ferment pyruvate into short-chain fatty acids (SCFAs; e.g., acetate, butyrate, and propionate), which may either support or disrupt epithelial function depending on concentration. (D) These microbial metabolic pathways present opportunities for intervention, including oleic acid to suppress dysbiotic anaerobes and lactic acid or *Lactobacillus*-based therapies to establish mucosal homeostasis and promote clearance of HPV-infected cells. The relative abundance of immune cells shown in the stroma is intended to conceptually reflect differences in local immune protection and mucosal homeostasis across the depicted states.

Dysbiotic communities actively convert amino acids into biogenic amines (e.g., putrescine, spermidine, and cadaverine), which can elevate vaginal pH (>4.5), impair mucosal immunity, and damage epithelial architecture [31,41,44,45]. These metabolic byproducts contribute to a permissive environment for hrHPV infection and persistence (Fig. 1). In addition, as a consequence of the viral immune evasion strategies, the amino acid source sustaining the survival of *Lactobacillus* species is greatly reduced, promoting an imbalance in the cervicovaginal microbiota [46]. Likewise, depletion of indole derivatives derived from microbial tryptophan metabolism, such as indole-3-acetic acid and indole-3-lactic acid, weakens aryl hydrocarbon receptor-mediated mucosal protection [47–50]. Furthermore, SCFAs such as acetate and butyrate, produced via microbial fermentation of pyruvate, can exert dual roles: at low concentrations, they help maintain epithelial function, but in excess (dysbiosis), they may disrupt epithelial barriers and fuel cytotoxic inflammation [35,51–55]. Nonetheless, associations between SCFAs and hrHPV infection outcomes are highly context dependent, varying with concentration, dominant producing taxa, vaginal pH, and the underlying inflammatory state [56].

Beyond biogenic amines and SCFAs, dysbiosis-driven shifts also affect metabolites involved in membrane integrity and host signaling. *Lactobacillus*-dominated, *Atopobiaceae*-negative profiles are relatively enriched in metabolites such as phosphoethanolamine and myoinositol, which are linked to epithelial membrane stability, phospholipid turnover, and cellular signaling (Table 1) [35]. In contrast, *Atopobiaceae*-associated dysbiosis is characterized by depletion of glycerophospholipids and enrichment of oxidative-stress-related metabolites and microbial-derived amines, reflecting a metabolically inflammatory microenvironment that may favor hrHPV persistence and lesion progression [35]. Other species, such as *Lactobacillus iners*, can lead to an accumulation of l-lactate in cervical tumors, resulting in therapy resistance in cervical cancer cells and metabolic rewiring [57]. These microbiome–metabolome interactions not only are markers of disease progression but may also be potential therapeutic targets [58]. Emerging interventions include the use of oleic acid to selectively suppress anaerobic bacteria and lactic acid-based gels or *Lactobacillus*-based probiotics to restore microbial and metabolic homeostasis [46,59–62]. Targeting metabolic dependencies, such as cysteine uptake in *L. iners*, also offers a precision-microbiome strategy to shift the ecosystem toward health (Fig. 1 [25]).

**Table 1.** Overview of cervicovaginal metabolomic studies across the HPV pathogenesis spectrum.

Study	Study design	HPV/disease stage	Sample size	Sample type	Analytical platform	Key alterations	Clinical/biological relevance
Borgogna *et al.* 2020 [31]	Cross-sectional	HPV− *vs* HPV+	*n* = 39	Vaginal self-swabs	LC–MS, GC–MS	Enrichment of biogenic amines and altered redox and lipid metabolism in HPV+	HPV infection and dysbiosis markers
Chorna *et al.* 2020 [36]	Cross-sectional	HPV− *vs* HPV+	*n* = 19	Vaginal swabs and urine	GC–MS	Increased levels of acetate, proline, and threonine and lower levels of succinate in HPV+	Noninvasive urine markers for HPV infection
Godoy-Vitorino *et al.* 2018 [73]	Cross-sectional	HPV− *vs* HPV+	*n* = 43	Cervical swabs and urine	GC–MS	Enrichment of 5-oxoproline, erythronic acid, *N*-acetylaspartate, and 4-hydroxybutyrate in HPV+	Noninvasive urine markers for HPV infection
Zhao *et al.* 2025 [120]	Cross-sectional	HPV− *vs* HPV+, with and without vaginitis	*n* = 164	Vaginal swabs	LC–MS	Increased levels of biogenic amines and altered lipids and lower levels of amino acids in HPV+ with vaginitis	Inflammation-dependent HPV metabolic signatures
Yang *et al.* 2025 [79]	Prospective cohort	HPV clearance *vs* persistent HPV, CIN2+	*n* = 56	Vaginal swabs	LC–MS	Enrichment of maltotriose, *N*-acetylaspartate, and *N*-acetylputrescine in HPV persistence	Microbiome–metabolome co-alterations linked to HPV persistence
Shen *et al.* 2025 [93]	Cross-sectional	HPV+ NILM, LSIL, or HSIL	*n* = 156	CVL	GC–MS	Increased levels of glucose-6-phosphate and erythrose and lower levels of succinate and sucrose in LSIL/HSIL	Lesion severity stratification
Dai *et al.* 2025 [91]	Cross-sectional	HPV+ CIN1, CIN2+	*n* = 43	Vaginal swabs	LC–MS	*Lactobacillus*-mediated enrichment of d-lactic acid, indole-3-lactic acid, amino acids in CIN1, and lipid shifts in CIN2+	Host–microbiome metabolic regulation across CIN stages
Pu *et al.* 2025 [56]	Cross-sectional	HPV−, HPV+, CIN1, CIN2+	*n* = 94	Cervicovaginal secretions	LC–MS	Increased levels of glycerophospholipids in CIN, bacteria–metabolite associations	Lesion-associated metabolic remodeling
Dai *et al.* 2026 [63]	Cross-sectional	LSIL, HSIL	*n* = 74	Vaginal swabs	LC–MS	Enrichment of amino acid metabolism, as well as d-lactic acid and indole-3-lactic acid in *Lactobacillus crispatus*-dominated communities and LSIL, compared to HSIL	*L. crispatus*-derived d-lactic acid modulation of HSIL risk
Bokulich *et al.* 2022 [69]	Cross-sectional	HPV−, HPV+, LSIL, HSIL, ICC	*n* = 72	CVL and vaginal swabs	LC–MS	Elevated sphingolipids, long-chain fatty acids, and 3-hydroxybutyrate in inflammation. Positive correlation of *N*-acetyl methionine sulfoxide with *L. crispatus*	Biomarker discovery for inflammation
Jimenez *et al.* 2024 [35]	Cross-sectional	HPV−, HPV+, LSIL, HSIL, ICC	*n* = 100	CVL and vaginal swabs	LC–MS	Increased levels of 4-hydroxybutyrate and sphingosine and lower levels of glycerophospholipids in *Atopobiaceae*-colonized women	*Atopobiaceae*-driven prooncogenic metabolism
Ilhan *et al.* 2019 [32]	Cross-sectional	HPV−, HPV+, LSIL, HSIL, ICC	*n* = 78	CVL and vaginal swabs	LC–MS	Enrichment of sphingolipids, 3-hydroxybutyrate, and xenobiotics in ICC and glutamine, pyroglutamine, and *N*-acetyltaurine in HPV−	Metabolic signatures of disease progression
Yu *et al.* 2024 [47]	Cross-sectional	HC, LSIL, HSIL, CC	*n* = 121	Vaginal swabs	LC–MS	Altered redox metabolism (glutathione-related), lipid remodeling, and amino acid perturbations with increasing lesion severity	Lesion progression and diagnostic markers
Kawasaki *et al.* 2024 [90]	Case–control	HPV− and HPV+ CIN1, CIN2, CIN3, or SCC	*n* = 399	Cervical mucus	LC–MS	Enrichment of malic acid, oxidized glutathione, and kynurenine in CIN2+/SCC	CIN2+/SCC triage biomarkers
Kawasaki *et al.* 2025 [102]	Cross-sectional, age-stratified	HPV− and HPV+ CIN1, CIN2, CIN3, or SCC	*n* = 296	Cervicovaginal swabs	LC–MS	Elevated cadaverine, 2-hydroxybutyrate, and TCA cycle intermediates in CIN3/SCC, particularly in premenopausal women; strong bacterial–metabolite correlations	Age-dependent microbiome–metabolome signatures
Paraskevaidi *et al.* 2020 [29]	Cross-sectional	HPV− *vs* HPV+, CIN1, CIN2, CIN3, CC	*n* = 130	Liquid-based cytology cell pellets	LA-REIMS	Altered phospholipids (PE, DG) in HPV+	Rapid screening and triage
Huang *et al.* 2025 [96]	Cross-sectional	HPV−, HPV+, LSIL, HSIL, CC	*n* = 40	Vaginal swabs	LC–MS	Altered amino acid pathways with lesion severity	Lesion stratification biomarkers
Ou *et al.* 2024 [95]	Cross-sectional	HPV+ NILM, CIN1, CIN2, CIN3, ICC	*n* = 100	CVL	LC–MS	Enrichment of oxidized glutathione, nucleotide metabolites, lipids, and organic acids in CIN3/ICC	Stage-dependent metabolic signatures of cervical lesion progression
Pu *et al.* 2025 [37]	Prospective cohort	HPV+ CIN1, CIN2+; HPV cleared *vs* persistent post-LEEP	*n* = 43	Cervicovaginal swabs	LC–MS	Elevated levels of acetate and altered glycerophospholipid levels in HPV clearance	Metabolic normalization with HPV clearance
Dai *et al.* 2024 [38]	Prospective cohort	Persistent HPV to CIN; posttreatment	*n* = 73	Vaginal swabs	LC–MS	Enrichment of glycerophospholipids and altered amino acid-related metabolites posttreatment	Metabolic remodeling after therapy
Du *et al.* 2025 [34]	Prospective longitudinal cohort	Persistent HPV to HPV clearance posttreatment	*n* = 32	Vaginal swabs	LC–MS	Early increase of glycerophospholipids, followed by later elevation in amino acid metabolism posttreatment	Metabolic recovery after treatment

hrHPV, high-risk human papillomavirus; LC–MS, liquid chromatography–mass spectrometry; GC–MS, gas chromatography–mass spectrometry; CIN1, CIN2, CIN2+, CIN3, cervical intraepithelial neoplasia grades 1, 2, 2+, and 3; NILM, negative for intraepithelial lesion or malignancy; LSIL, low-grade squamous intraepithelial lesion; HSIL, high-grade squamous intraepithelial lesion; CC, cervical cancer; PE, phosphoethanolamine; DG, diglyceride; CVL, cervicovaginal lavage; ICC, invasive cervical carcinoma; SCC, squamous cell carcinoma; TCA, tricarboxylic acid; LA-REIMS, laser-assisted rapid evaporative ionization mass spectrometry; LEEP, loop electrosurgical excision procedure; HPV− denotes the absence of detectable hrHPV DNA at sampling and does not exclude latent infection or viral loads below the assay detection limit; HC indicates clinically healthy controls as defined by the original study

Overall, the relationship between hrHPV persistence and the cervicovaginal metabolome appears bidirectional. Microbiota-derived metabolites can directly modulate viral outcomes; for example, *Lactobacillus*-associated metabolites such as d-lactic acid are enriched in women who clear infection, while persistent hrHPV infection reshapes the metabolic landscape through host transcriptional changes that influence both host and microbial metabolism [39,63]. Recent studies further suggest that dysbiotic taxa and their associated metabolites may mediate suppression of protective *Lactobacillus* communities during persistence [46]. Together, these observations support a feedback loop in which metabolic dysregulation and viral persistence reinforce each other.

## The Cervicovaginal Metabolome in Health and HPV-Negative States

A metabolically healthy cervicovaginal environment reflects a biochemical landscape that supports epithelial barrier integrity, controlled immune activation, and redox balance, thereby maintaining mucosal resilience against physical, microbial, and inflammatory perturbations [24,64]. Core features of this state include the predominance of organic acids (notably lactic acid), intact carbohydrate metabolism with adequate glycogen availability, balanced lipid and phospholipid composition, and sufficient antioxidant capacity [24,51]. Together, these features contribute to a low vaginal pH, preservation of the mucus layer, and reinforcement of epithelial tight junctions, limiting pathogen adherence and excessive immune activation.

In the context of cervicovaginal biology, an HPV-negative state should not be compared with a universally healthy or homeostatic microenvironment. HPV negativity reflects the absence of detectable viral DNA at the time of sampling but may coexist with metabolic and microbial perturbations driven by bacterial vaginosis, vulvovaginal candidiasis, sexually transmitted infections, hormonal fluctuations, or inflammatory conditions. A hallmark of an HPV-negative state is the predominance of lactic acid, glutathione, phospholipids, fatty acids, glycogen, and cytosine, all of which contribute to epithelial barrier maintenance, redox balance, and immune readiness [39,59,65,66]. For example, Borgogna *et al.* [31], by performing metabolomics on self-collected, mid-vaginal swabs, reported that HPV-negative American women, particularly those with *Lactobacillus*-dominated microbiomes, had high levels of glycogen and phospholipids, which align with a thick, protective mucus layer (Table 1) [23,67,68]. Bokulich *et al.* [69], by analyzing CVL samples, also found these metabolites enriched in HPV-negative Caucasian and Hispanic women, further supporting the role of intact lipid and carbohydrate metabolism in maintaining cervicovaginal health [23,67,70]. Overall, these findings suggest that the healthy and HPV-negative cervicovaginal microenvironment is characterized by energy-efficient, anti-inflammatory, and structurally intact metabolic processes, which are likely to contribute to cervical health and facilitate rapid viral clearance.

The metabolically healthy cervicovaginal environment is characterized by a diverse set of metabolites that are repeatedly reported across studies and that serve as key indicators of epithelial integrity, immune regulation, microbial activity, and redox balance. These include organic acids such as lactic acid and acetate, which maintain vaginal acidity and modulate epithelial and immune function [21,71]; biogenic amines and polyamines, generated through microbial amino acid decarboxylation and associated with elevated pH and inflammation when enriched [41,44]; amino acids and N-acetylated derivatives reflecting protein turnover, immune activation, and epithelial repair [49,64]; lipids and glycerophospholipids involved in membrane integrity and signaling [72]; carbohydrates and glycolytic intermediates supporting epithelial metabolism and microbial growth [64,67]; redox-related metabolites such as glutathione indicating oxidative stress responses [31,32]; and microbially derived metabolites including indole derivatives and SCFAs that mediate host–microbe signaling [22,49–51]. Importantly, the biological interpretation of these metabolites is highly context dependent and influenced by the sample matrix, microbial composition, and disease stage. Together, these metabolites provide a baseline framework for understanding how metabolic networks are progressively altered during HPV acquisition, persistence, cervical lesion development, and cervical carcinogenesis.

## Metabolic Disruption and Dysbiosis during HPV Infection before Persistence and Lesion Development

**Fig. 2.**
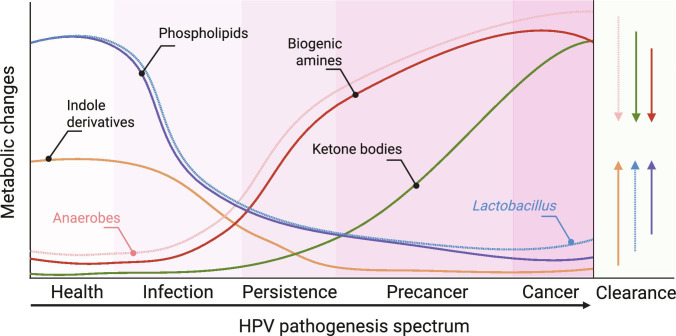
Temporal trends of key metabolic and microbial features across the HPV pathogenesis spectrum. This figure illustrates the dynamic changes in selected cervicovaginal metabolites and microbial signatures from HPV-negative health through HPV infection and persistence, precancerous lesions, cervical cancer, and viral clearance. Viral clearance is depicted as a potential metabolic state that may occur at multiple stages of infection and is not intended to represent a chronological outcome following cancer. Protective features such as phospholipids, indole derivatives, and *Lactobacillus* (*Lactobacillus crispatus*, *Lactobacillus jensenii*, or *Lactobacillus gasseri*) decrease progressively with disease advancement, which may be restored upon clearance at any stage. In contrast, biogenic amines, ketone bodies (e.g., hydroxybutyrate), and anaerobic bacteria (e.g., *Gardnerella*, *Prevotella*, and *Fannyhessea*) increase during persistence and carcinogenesis, reflecting dysbiosis, inflammation, and altered energy metabolism. Trends are synthesized from multiple metabolomics and microbiome studies and illustrate potential biomarkers and therapeutic targets for HPV-related cervical disease.

Upon HPV infection, even in the absence of cytological abnormalities, initial metabolic shifts signal a breakdown in mucosal equilibrium [46]. A recurring observation across studies is the rise in biogenic amines, notably putrescine, cadaverine, tyramine, and their intermediates [31,69]. These compounds are products of microbial amino acid decarboxylation, whereby anaerobic or facultative anaerobic bacteria convert ornithine, lysine, and tyrosine into polyamines and related amines, a process commonly enriched in dysbiotic cervicovaginal communities (Figs. 1 and 2) [45]. The presence of these amines correlates with a shift from *Lactobacillus*-dominated states to more diverse or anaerobic microbiota, suggesting microbial metabolism as an early driver of biochemical disruption [24,44,46].

Parallel to microbial shifts, HPV infection is also associated with depletion of host-derived protective metabolites. Chorna *et al.* [36] and Godoy-Vitorino *et al.* [73], both performing metabolomics on urine samples, reported elevated acetate and tricarboxylic acid (TCA) cycle intermediates (e.g., aconitic acid and citric acid) in HPV-positive Puerto Rican women, suggesting potential systemic metabolic correlates of genital tract alterations associated with HPV infection (Table 1) [40,71,74]. However, these findings were derived from relatively small cohorts and a systemic sample matrix and therefore require independent validation before broader conclusions can be drawn. These shifts may reflect increased energy metabolism and possible mitochondrial stress [75]. In Ilhan *et al.* [32], HPV-positive Hispanic and non-Hispanic women without cervical lesions showed decreased levels of glutamine, pyroglutamine, and *N*-acetyltaurine, which are metabolites involved in amino acid turnover, nucleotide metabolism, and mucosal repair (Table 1) [76–78]. This suggests that during an initial HPV infection, there may be a burden on the local immune and metabolic networks.

## The Metabolic Cost of HPV Persistence and Clearance

Persistent hrHPV infection introduces a deeper metabolic burden on the cervicovaginal microenvironment. Yang *et al.* [79], by examining vaginal swabs from Chinese women with persistent HPV and high-grade CIN compared to those who cleared the virus, revealed marked elevations in N-acetylated amino acids (e.g., *N*-acetylputrescine and *N*-acetylaspartate) and maltotriose in persistent cases (Table 1). These metabolites point to chronic immune activation, membrane remodeling, and changes in cellular signaling pathways [23,24,80]. Across studies, N-acetylated amino acids repeatedly emerge as markers of chronic HPV infection and persistence, likely reflecting sustained amino acid turnover, redox stress, and host–microbial metabolic adaptation [73,79]. Pu *et al.* [37], by analyzing vaginal secretions from Chinese women who failed to clear HPV after a loop electrosurgical excision procedure, reported a similar metabolic pattern showing altered glycerophospholipids [72].

Alternatively, those who cleared HPV showed enriched levels of acetate, glycerophospholipids, and several lipid intermediates potentially involved in tissue repair and anti-inflammatory responses [37,51,72]. Extending these observations, Du *et al.* [34] analyzed vaginal swabs from a relatively small cohort of Chinese women and performed a 1-year longitudinal multiomics analysis following treatment for cervical precancer, demonstrating that restoration of vaginal health is characterized by coordinated normalization of both microbiome composition and metabolite profiles. Notably, metabolomic recovery was marked by an early increase of glycerophospholipids and restoration of amino acid homeostasis [34]. Importantly, glycerophospholipid enrichment may reflect distinct biological processes depending on context, including membrane repair and epithelial restitution during clearance, versus inflammatory membrane turnover and oncogenic remodeling in high-grade lesions (Table 1). Yang *et al.* [79] also found that women who cleared HPV had elevated levels of indole derivatives, dl-carnitine, and tryptophan, possibly reflecting the well-documented transitional dysbiosis state post-viral clearance, which is a temporary, metabolically unstable microbiome–metabolome configuration in the cervicovaginal microenvironment [81,82], highlighting how metabolomics may help differentiate productive antiviral responses. Interestingly, Jimenez *et al.* [35] observed that women with persistent HPV, especially those enriched with *Atopobiaceae* species, such as *F. vaginae*, *Fannyhessea massiliense*, *Fannyhessea species type 2*, and *Lancefieldella deltae*, had increased xenobiotics (e.g., catechol sulfate and quinate) and decreased phosphoethanolamine and myoinositol, further implicating microbial-derived inflammation and membrane instability [83].

Nonetheless, the transition from transient infection to persistence is not clearly delineated in all studies. While some data suggest a linear continuum of metabolic dysregulation, others indicate a more punctuated shift characterized by the collapse of antioxidants and immune defenses. Moreover, variability in sample matrices and follow-up times complicates the interpretation of these results. Taken together, these findings support the hypothesis that metabolomics, when integrated with microbial and immunological profiles, may offer predictive markers of persistence risk and therapeutic targets to modulate the local environment toward viral clearance [24,27,84,85].

## Cervical Lesions and Metabolic Deterioration

With the progression of persistent HPV infections toward cervical lesions, integrated molecular profiles reflect a state of progressive inflammation, oxidative damage, and epithelial stress [8,86,87]. In this context, multiomics approaches typically integrate cervicovaginal metabolomics with microbiome profiling (to capture dysbiosis and microbial metabolic potential), transcriptomics or proteomics (to assess epithelial stress responses, inflammation, and oncogenic signaling), and immunological readouts (to quantify local immune activation), enabling a more comprehensive characterization of lesion-associated microenvironmental changes [13,88]. Ilhan *et al.* [32] and Bokulich *et al.* [69] reported declining levels of NAD^+^, cytosine, and glutamine in women with LSIL and HSIL, suggesting compromised redox balance and impaired nucleotide and energy metabolism [76,89]. These changes co-occurred with increasing levels of sphingolipids, biogenic amines, and TCA cycle intermediates like 3-hydroxybutyrate, indicating both microbial dysbiosis and host metabolic stress (Fig. 2) [32,69,90].

Consistent with these observations, Pu *et al.* [56] reported progressive cervicovaginal metabolomic remodeling across HPV infection and CIN grades, characterized by lipid enrichment and depletion of amino acid and nucleotide metabolites in high-grade lesions. Likewise, Kawasaki *et al.* [90], by analyzing cervical mucus from Japanese women with low- and high-grade CIN, revealed progressive increases in TCA cycle intermediates, kynurenine, and oxidized glutathione from CIN to cervical cancer (Table 1). Dai *et al.* [91], by examining vaginal swabs from Chinese women, also reported that CIN grade 1 (CIN1) lesions retain *Lactobacillus*-associated metabolites, including d-lactic acid and indole-3-lactic acid, whereas CIN grade 2+ (CIN2+) is marked by lipid remodeling and attenuation of these protective pathways, reinforcing a staged metabolic deterioration during cervical carcinogenesis. Although CIN grade is associated with distinct host transcriptomic programs, metabolic variation appears more strongly structured by *Lactobacillus* dominance than by lesion severity, suggesting that microbiome composition may outweigh epithelial transformation in shaping the cervicovaginal metabolome [56,91].

Yu *et al.* [47], by assessing vaginal secretions from Chinese women, further demonstrated that lesion severity correlated with higher levels of oxaloacetic acid, abrine, and glutathione, alongside lower levels of antioxidants such as dehydroascorbate and glutathionylspermidine, a signature of metabolic decompensation (Table 1). The increased glutathione levels may reflect total or oxidized glutathione pools, likely representing compensatory antioxidant responses to increased oxidative stress or altered epithelial metabolism during lesion progression [90,92]. However, reduced glutathione (GSH) and oxidized glutathione (GSSG) ratios (GSH/GSSG), which provide a more informative measure of redox status, were generally not reported in these studies. In addition, Shen *et al.* [93], by analyzing CVL from Chinese women, reported significant increases in glucose-6-phosphate, gluconic acid lactone, and rare amino acid derivatives such as dl-*p*-hydroxylphenyllactic acid and 2,3-dihydroxypyridine in HSIL compared to LSIL [94]. Meanwhile, their observed reductions in sucrose, succinate, and amino acids such as gamma-aminobutyric acid and spermidine suggest impaired energy generation and cellular repair [93]. Together, these alterations indicate an energy-imbalanced and pro-inflammatory microenvironment, with early rewiring of glycolytic and glycogen-related pathways that intensifies with lesion severity and reflects compromised epithelial metabolic homeostasis despite increased metabolic flux [79,93]. Ou *et al.* [95] confirmed this trend on CVL from Chinese women, showing declining levels of protein digestion and carbohydrate metabolism metabolites from CIN to ICC, while oxidative stress markers increased. By assessing vaginal swabs from Chinese women, Huang *et al.* [96] also identified lipid remodeling and amino acid pathway dysregulation as hallmarks of lesion progression (Table 1). Nevertheless, some of these reported metabolite alterations, such as gluconic acid lactone, 3-isochromanone, abrine, dl-*p*-hydroxylphenyllactic acid, and 2,3-dihydroxypyridine, have unclear or poorly established relevance in the cervicovaginal microenvironment [97]. Their detection may reflect technical artifacts, misannotation, or metabolites of exogenous origin and should be interpreted cautiously until validated in independent datasets or functionally contextualized in HPV-related cervical pathology [93,95,96].

A prospective study by Dai *et al.* [38] evaluated vaginal swabs from Chinese women undergoing surgical removal of high-grade CIN and demonstrated marked changes in metabolite profiles 6 months posttreatment, despite minimal shifts in microbial composition. The authors described an enrichment of glycerophospholipids and depletion of organic nitrogen compounds and amino acid derivatives, indicating a restoration of metabolic homeostasis following viral and lesion clearance (Table 1) [37]. Meanwhile, Paraskevaidi *et al.* [29], using LA-REIMS on liquid-based cytology-derived cell pellets from UK-based women, identified elevated ceramides and complex phospholipids as the most discriminatory features distinguishing hrHPV infection and high-grade cervical disease, reinforcing the role of lipid remodeling in advanced disease [32,48,69,98].

A key limitation across studies, nevertheless, lies in the inconsistent categorization of lesions, often using Bethesda or histological classifications, grouping CIN2 and CIN3 or LSIL/HSIL under the umbrella terms “CIN2+” or “SIL”, respectively, which may mask stage-specific metabolic patterns [3]. Moreover, the cross-sectional nature of most studies precludes inference about whether the observed shifts drive lesion development or result from it (Table 1). In general, the convergence of findings across cohorts supports the notion that progressive lesion severity is metabolically traceable, offering opportunities for early cancer detection and triaging of HPV-positive women.

## Metabolic Reprogramming in Cervical Cancer

Cervical cancer marks a metabolic state of full transformation, mirroring hallmarks of cancer biology such as altered bioenergetics, immune evasion, increased glycolysis, and enhanced biosynthesis [65,66,94,99–101]. While cervicovaginal metabolomic studies capture the metabolic consequences of these processes in patient samples, mechanistic insights into the underlying pathways have also been derived from cervical cancer cell line models [47]. Across multiple studies [32,47,69,95,102], cervical cancer is characterized by significantly elevated levels of 2/3-hydroxybutyrate, long-chain fatty acids, adenosine monophosphate, aconitic acid, and sphingolipids (Table 1 and Fig. 2). These molecules reflect increased fatty acid oxidation, ketone body production and mitochondrial dysfunction processes that enable tumor cells to sustain proliferation under oxidative stress [48,52,103,104]. Additionally, several nucleotide and purine metabolites seemingly increase, indicating shifts in nucleotide biosynthesis and redox signaling. At the same time, cancer samples showed consistent depletion of metabolites essential for membrane stability and mucosal defense, such as glycogen, glutathione, cytosine, and phosphoethanolamine [24,41]. The observed reductions in myoinositol and glycerophospholipids further support the hypothesis of epithelial degradation and metabolic exhaustion [35]. Ou *et al.* [95] also identified increased levels of toxic xenobiotics like aflatoxin B1 and 4-hydroxydebrisoquine in ICC, hinting at impaired detoxification capacity or increased environmental susceptibility [35,95,105].

## Multiomics Integration and Computational Modeling of the Cervicovaginal Ecosystem

The cervicovaginal microenvironment is a complex system in which microbial communities, host epithelial and immune responses, and metabolic outputs are tightly interconnected. Metabolomics provides a functional snapshot of this ecosystem but does not fully capture the mechanisms underlying HPV persistence, clearance, or progression. Integrating metabolomic data with complementary omics layers, including microbiome profiling, transcriptomics, proteomics, and immunological measurements, enables a systems-level view of these interactions [35,55,69,106]. Such approaches consistently show that metabolic profiles often correlate more strongly with clinical outcomes than microbial composition alone, supporting the idea that metabolites reflect the combined activity of host and microbiome processes. For example, Bokulich *et al.* [69] identified metabolites such as *N*-acetyl methionine sulfoxide, sphingolipids, and 3-hydroxybutyrate as key predictors of cervicovaginal microenvironmental states, outperforming microbial composition alone in several predictive models.

Computational and integrative frameworks are beginning to reveal the structure and directionality of these interactions. Network-based analyses can identify clusters of co-occurring microbes and metabolites that define distinct ecological states, while mediation models highlight metabolites that link microbial composition to host responses or disease phenotypes [54,63,66,79,91,107]. For instance, Yang *et al.* [79] identified coordinated associations between persistent HPV infection, *N*-acetylputrescine, maltotriose, and specific bacterial taxa, whereas Dai *et al.* [63] linked *Lactobacillus*-associated metabolites such as d-lactic acid and indole-3-lactic acid to cervical gene expression and reduced risk of precancerous lesions. These approaches are particularly relevant in HPV infection, where microbial effects are likely mediated through metabolic pathways. Machine learning models integrating metabolomic, microbial, and clinical features further support risk stratification, with metabolite-based features frequently emerging as strong predictors [32,69,108]. Of note, sphingolipids, 3-hydroxybutyrate, *N*-acetyl methionine sulfoxide, and several glycerophospholipid species have repeatedly been associated with inflammation, disease severity, or HPV-related outcomes across integrative analyses [37,38,63,69,91].

Multiomics integration also offers opportunities to bridge local and systemic biology. Metabolic alterations associated with HPV infection are detectable not only in cervicovaginal samples but also in systemic matrices such as urine, supporting the development of noninvasive biomarkers [33,36,109]. However, variability in sampling, analytical platforms, and data processing remains a major challenge for reproducibility. Standardized workflows and validated computational frameworks will be essential to move from associative findings to clinically actionable insights [110]. Emerging community efforts, including standardized reporting guidelines, quality-control initiatives, public repositories such as MetaboLights, and expanding tandem mass spectrometry spectral libraries, may help improve reproducibility, metabolite annotation, and cross-study comparability [111,112]. Taken together, integration of microbiome and metabolome data provides a foundation for identifying key regulatory pathways and advancing precision strategies for HPV-related disease prevention and management. These interactions also reflect broader host–environment influences, including lifestyle, exposures, and systemic metabolism, emphasizing the interconnected nature of local and systemic biology [113–117].

## Methodological Limitations and Translational Perspectives

Despite increasingly consistent trends across studies, several methodological limitations currently constrain the reproducibility and clinical translation of cervicovaginal metabolomic findings in HPV-related disease. A major challenge lies in the heterogeneity of sample matrices, including vaginal swabs, CVL, vaginal secretions, cytological samples, and urine, each of which captures distinct biological compartments and metabolic contributions from host tissues, resident microbes, and systemic metabolism [29,31,32,90]. As a result, metabolite profiles are not directly comparable across studies, and conclusions drawn from one matrix may not readily generalize to another. In addition, differences in analytical platforms, most commonly LC–MS and GC–MS, may introduce platform-specific biases in metabolite coverage, sensitivity, and annotation, further complicating cross-study validation and meta-analysis.

Most available studies are cross-sectional and exploratory, limiting causal inference and the ability to distinguish metabolic drivers of HPV persistence or progression from downstream consequences of disease. Cohort sizes are often modest, lesion categories are inconsistently defined, and important confounders such as co-infections, hormonal status, age, sexual practices, and baseline microbiome composition are not uniformly controlled [64,102]. In addition, the current evidence is geographically concentrated, with many metabolomic studies originating from Chinese cohorts and comparatively fewer investigations from European, African, Latin American, and other populations, potentially limiting the generalizability of reported metabolic signatures across diverse populations and healthcare settings [118,119]. Importantly, several metabolomic alterations attributed to HPV infection overlap with signatures observed in bacterial vaginosis, inflammation, and other genital infections (e.g., *Mycoplasma hominis*), highlighting co-infection and baseline microbiome composition as key confounders [107,120,121]. Furthermore, metabolite identification remains a critical bottleneck, with several reported features lacking definitive structural annotation or functional contextualization within the cervicovaginal niche [91]. Together, these factors hinder biomarker reproducibility and underscore the need for standardized sampling protocols, harmonized analytical pipelines, and longitudinal study designs.

Notably, current interventions for HPV-associated cervicovaginal disease predominantly target epithelial repair and microbiome restoration [122,123]. Several vaginal therapies are already commercially available for vaginal health indications or have been evaluated in interventional clinical studies, including formulations containing *Coriolus versicolor*-derived compounds or carboxy-methyl-β-glucan [122,123]. These studies have reported improvements in cervical epithelialization, vaginal pH normalization, vaginal health indices, and increased rates of cytological regression or HPV clearance compared with controls. Importantly, however, none of these trials incorporated metabolomic profiling or predefined metabolic endpoints, and no metabolome-based diagnostics or metabolite-targeted therapies are currently approved for HPV-associated disease [60,122–124]. The clinical benefits observed in these studies are therefore best interpreted as indirect metabolic effects arising from improved mucosal integrity, immune modulation, and shifts in vaginal microbiome composition.

From a translational perspective, these limitations also define clear opportunities. Longitudinal, multicenter studies integrating metabolomics with microbiome, immunological, and clinical data are essential to establish robust, stage-specific metabolic signatures predictive of HPV acquisition, persistence, clearance, or progression. Functional validation of recurrent metabolite classes, such as biogenic amines, N-acetylated amino acids, glycerophospholipids, and redox-associated metabolites, will be critical to move beyond association toward mechanistic insight [39,57,125,126]. Importantly, the relative stability of metabolic outputs compared to taxonomic microbial profiles suggests that metabolomics may offer a more tractable route to noninvasive diagnostics, particularly when applied to scalable matrices such as urine or self-collected vaginal samples [31,36,69]. Emerging analytical frameworks, including mediation-based multiomics approaches, are beginning to identify specific microbial taxa and metabolites as intermediates linking host gene expression to HPV outcomes, providing early evidence toward causal inference [38,63,91]. Ultimately, overcoming current methodological fragmentation will be key to translating cervicovaginal metabolomics from descriptive profiling into clinically actionable tools for HPV risk stratification, triage, and precision prevention.

## Conclusion

This review synthesizes current evidence demonstrating that cervicovaginal metabolic alterations closely track the natural history of HPV infection and cervical carcinogenesis. Functionally healthy cervicovaginal environments are characterized by metabolic features that support epithelial integrity, redox balance, and immune homeostasis, whereas HPV infection disrupts this equilibrium through oxidative stress and the accumulation of microbially derived metabolites. These disruptions intensify with viral persistence and lesion development, culminating in cervical cancer marked by extensive metabolic reprogramming involving lipid biosynthesis, energy metabolism, and nucleotide turnover, consistent with hallmarks of oncogenic transformation. Metabolic signatures provide functional insight into host–microbiome interactions, capturing coordinated biological processes that extend beyond taxonomic composition alone. Recurrent alterations in biogenic amines, N-acetylated amino acids, sphingolipids, and redox-associated metabolites highlight key pathways implicated in HPV-related disease, despite methodological heterogeneity. Looking forward, a systems-level integration of metabolomics with microbiome, host, and clinical data will be essential to move from descriptive associations toward mechanistic and clinically actionable insights. Advances in multiomics integration and computational modeling are expected to improve risk stratification, enable noninvasive diagnostics, and support precision prevention strategies [38,66,106,127,128]. These approaches may also capture broader microenvironment interactions relevant to disease risk. Such developments are particularly relevant for low-resource settings, where scalable, metabolite-based tools could meaningfully reduce the global burden of HPV-associated cervical cancer [129].
